# European Health Data Space—An Opportunity Now to Grasp the Future of Data-Driven Healthcare

**DOI:** 10.3390/healthcare10091629

**Published:** 2022-08-26

**Authors:** Denis Horgan, Marian Hajduch, Marilena Vrana, Jeannette Soderberg, Nigel Hughes, Muhammad Imran Omar, Jonathan A. Lal, Marta Kozaric, Fidelia Cascini, Verena Thaler, Oriol Solà-Morales, Mário Romão, Frédéric Destrebecq, Edith Sky Gross

**Affiliations:** 1 European Alliance for Personalised Medicine, 1040 Brussels, Belgium; 2 Department of Molecular and Cellular Engineering, Jacob Institute of Biotechnology and Bioengineering, Faculty of Engineering and Technology, Sam Higginbottom University of Agriculture, Technology and Sciences, Prayagraj 211007, India; 3 Institute of Molecular and Translational Medicine, Faculty of Medicine and Dentistry, Palacký University, 77900 Olomouc, Czech Republic; 4 European Heart Network, 1000 Brussels, Belgium; 5 Juvenile Diabetes Research Foundation, 68 Rue du Faubourg Saint Honoré, 75008 Paris, France; 6 Janssen Pharmaceutica NV, 2340 Beerse, Belgium; 7 Academic Urology Unit, Health Sciences Building, University of Aberdeen, Aberdeen AB25 2ZD, UK; 8 Institute for Public Health Genomics, Department of Genetics and Cell Biology, GROW School of Oncology and Developmental Biology, Faculty of Health, Medicine and Life Sciences, Maastricht University, 6229 ER Maastricht, The Netherlands; 9 Section of Hygiene and Public Health, Department of Life Sciences and Public Health, Catholic University of the Sacred Heart, 00168 Rome, Italy; 10 MedTech Europe, 1000 Brussels, Belgium; 11 FHiTT-Fundació HiTT, 08015 Barcelona, Spain; 12 Intel Corporation, 2550 Kontich, Belgium; 13 European Brain Council, 1000 Brussels, Belgium; 14 EURORDIS-Rare Diseases Europe, 75014 Paris, France

**Keywords:** European, health, data, space, healthcare, policy framework, personalised medicine, data protection, governance, access to data, governance, EU values, diagnosis, treatment, prevention, health policy

## Abstract

The May 2022 proposal from the European commission for a ‘European health data space’ envisages advantages for health from exploiting the growing mass of health data in Europe. However, key stakeholders have identified aspects that demand clarification to ensure success. Data will need to be freed from traditional silos to flow more easily and to cross artificial borders. Wide engagement will be necessary among healthcare professionals, researchers, and the patients and citizens that stand to gain the most but whose trust must be won if they are to allow use or transfer of their data. This paper aims to alert the wider scientific community to the impact the ongoing discussions among lawmakers will have. Based on the literature and the consensus findings of an expert multistakeholder panel organised by the European Alliance for Personalised Medicine (EAPM) in June 2022, it highlights the key issues at the intersection of science and policy, and the potential implications for health research for years, perhaps decades, to come.

## 1. Introduction

The proposal represents a huge opportunity for enabling better use of the vast amounts of health data that are currently neglected—crucially improving access for researchers and public health authorities to large-scale data sets for secondary use. Correctly implemented, this ambitious concept could lead to better diagnosis and treatment; improved patient safety and continuity of care; greater healthcare efficiency; and enhanced opportunities for research and innovation. This article offers an alert in a bid to ensure that the final form of the legislation will not hinder but will assist the scientific/research/medical community. It is a translational policy piece from the multistakeholder community (Health Care Professionals (HCPs), patients, industry, payers, as well as institutional partners) offering policy guidance at pan-EU, country, and regional levels on how the opportunities can be seized—and cautioning against neglect of issues that might otherwise cause the opportunities to be lost.

### 1.1. Data Governance

For a paper offering guidance to different healthcare actors and the policy community, it is important at the outset to establish the context in which the European Health Data Space (EHDS) is being created, and particularly the context for data governance and the issues around this complex and highly sensitive issue [1,2].

Data governance means different things to different stakeholder groups—as is to be expected in an era where the options for data collection and use, as well as the sheer volumes and speed of possible analysis, are growing exponentially [3]. For campaigners for patients’ rights, data governance often means strict control by each individual over personal data and its transmission or use elsewhere—an essentially protective concept [4,5,6]. At the other end of the spectrum, the emphasis is more on data governance as a way of ensuring optimal use of a valuable resource in a manner compatible with protection of data sources [7,8]. In between is a range of intermediate positions with more or less emphasis on either protection or exploitation, and with a focus on more technical aspects of governance—for instance, on ensuring data is of high quality, or compliant with agreed standards, or maintained in a secure environment. In this context, it can mean the process of managing the availability, usability, integrity, and security of the data in systems, based on internal data standards and policies that control data usage. Effective data governance, in this sense, ensures that data is consistent and trustworthy and is not misused [9,10,11,12].

Within all the variants of approaches to data governance, the terms which are used are subject to interpretation, complicating the discussions further [13,14,15]. In discussing “use”, different stakeholders do not always clarify what use, by whom, for what purpose, and subject to what conditions. In discussing privacy, they do not always explain whether privacy is absolute, or indeed where ownership of data actually lies—or whether it has limits. Terms such as quality and standards are not always defined. However, what is evident is that critical issues such as the collection, storage, use, and ownership of data continue to increase in volume and sophistication. So, on the one hand, data analytics are increasingly offering new ways to help optimise operations and drive decision-making, while on the other hand, regulatory and public pressure on privacy issues continue to gather strength [16,17]. 

### 1.2. Stakeholder Influence

Over 2023 and probably 2024, intensive discussions among policymakers at the national and European level will shape the final form of the legislation through the EU’s extensive law-making mechanisms and will also guide the way that member state governments then implement the legislation in detail at national level. During this period, the views that policymakers adopt are open to influence by all stakeholders, including the science and research community. As in any breakthrough area—and data may be the epitome of breakthrough areas—policymaking is faced with trying to figure out, in terms of governance, how best to balance competing needs, in this case the protection of data with the prospect of its innovative use. Insufficient protection could damage the flow of data at its source, cutting off invaluable resources [18]. However, data protection that is so complete that it prevents the acquisition and effective use of data could equally damage the flow of innovation.

For the EHDS, there is the possibility of a rational discussion with experts so that the EU as a region can move forward collectively to construct a supranational data governance structure that makes sense but does not require constant subsequent adjustment to remedy its excesses or deficiencies, with consequent harm to patients, data use, and innovation [1,19,20,21,22]. This is what makes early and constructive input to the discussions from all concerned stakeholders—and particularly the scientific and research community—important.

## 2. What Is EHDS?

EHDS is a part of the EU’s overall strategy on data policy, a context that includes the Data Governance Act and the draft Data Act, and also takes account of world-level data [23,24,25]. EHDS is designed to complement other EU legislation, including the EU General Data Protection Regulation (“GDPR”), the Medical Device Regulation (“MDR”), and the In Vitro Diagnostics Regulation (“IVDR”)—and has the significant difference that it applies to a specific sector, whereas the other EU data legislation to date is all generic, with provisions of a horizontal character [2,26] —likely to be augmented with legislation on artificial intelligence and cybersecurity. EHDS is aimed solely at health and is recognised as a potential step-change in EU health policy by legislators and a wide range of stakeholders, from patient groups, academics, and civil society organisations to the healthcare industry. The EU ambitions for the legislation are summarised in Box 1.

Box 1 **Summarised review of what the EHDS could provide to different stakeholders according to the European Commission.**

According to the European Commission, EHDS will provide immediate and easy access to data in electronic form, free of charge, allowing sharing with health professionals across the EU to improve health care delivery. Citizens will be in full control of their data and can restrict access and obtain information on how their data are used and for which purpose. Patient summaries, ePrescriptions, images and image reports, laboratory results, and discharge reports will be issued and accepted in a common European format, with mandatory interoperability and security. Citizens’ rights will be safeguarded by new digital health authorities in each member state, backed by a cross-border digital infrastructure (MyHealth@EU) supporting patients in sharing their data.

EHDS will also, the Commission continues, improve the use of health data for research, innovation, public health, policymaking, and regulatory purposes, within a new legal framework that will allow access—under strict conditions—for researchers, innovators, public institutions, or industry. Access will be granted only if the requested data are to be used for specific purposes, in closed, secure environments, and without revealing the identity of the individual. New national health data access bodies will be connected to a decentralised EU-infrastructure for secondary use (HealthData@EU) to support cross-border projects [1].

The June 2022 EU Health Council reflected this significance. The Council Presidency identified the subject of digital as an essential pillar of the creation of an effective European Health Union. The draft regulation “aims to unlock the full potential of health data for all individuals, patients, innovators and public decision-makers” [27,28]. 

The timing of the proposal is apt, with momentum developed by recent EU experience: “The COVID-19 pandemic has demonstrated the need to ensure rapid and secure access to health data in order to ensure the continuity and quality of care pathways. The health data that have been produced and shared have demonstrated their full potential for emergency decision-making, patient care and monitoring, research and innovation at national, European and international level, and the development of statistics and public policies,” according to the Presidency [27]. The experience has “demonstrated Europe’s capacity to provide innovative solutions and to turn them into an unrivalled international standard.” Kyriakides takes the same view, endorsing the need for courageous action: “It was bold, coordinated decision-making during the pandemic that saw us deliver digital health solutions rapidly, together” [29]. 

The timing of the proposal is also apt in that it places Europe at the forefront of attempts to intelligently regulate the sharing of health data—a tangled issue that has so far received only limited and piecemeal treatment in other jurisdictions and regions. It offers a lead that could—as has happened in many other areas—see EU rules inspire international guidance. It is also particularly helpful at a time when the UK, now separated from the EU in this as in many other respects, is still feeling its way toward its own rules. There are obvious merits in maximum alignment of regulation among economies so closely linked.

Europe’s main drug industry organisations view EHDS favourably. The European Federation of Pharmaceutical Industries and Associations (EFPIA) leadership announced it is “delighted to see Europe moving forward in the critical area of health data”, pledging “strong support” for the project’s dual aims. It described the conjunction of recent EU legislation and new proposals—on EHDS, data governance, network and information security, and data protection—as “an unprecedented opportunity to shape the future health data and digital ecosystem.” Additionally, both Europe and Medicines for Europe see great potential [23].

The European Patients Forum saw EHDS as an “ambitious and timely initiative”, endorsing the EU’s view that harnessing the power of health data can help deliver better care and improve patients’ lives [30]. EURORDIS-Rare Diseases Europe, the nonprofit alliance of rare disease patient organisations, welcomes the EHDS focus on health-data control [31]. AIM, the international grouping of mutual insurance organisations, welcomed the launch of the proposal and said it is “looking forward to interesting discussions.” In particular, it sees the initiative as “good news for cross-border healthcare.” The digital industry grouping, Digital Europe, gave immediate support for “this decisive step towards a harmonised internal market for health” [32]. MedTech Europe welcomed the Commission’s intention to create an enabling environment for health-data sharing in the EU and stated that the proposed EHDS needs to address barriers to data sharing, advance investment in infrastructure, foster the adoption of international interoperability standards, and build trust in health-data sharing [33].

Pragmatic approaches have already demonstrated the benefits of sharing data [34,35,36,37]. The fight against rare diseases has spearheaded the pooling of resources and knowledge for better patient care in the EU, with enhanced cooperation on shared principles leading to increased ability to provide healthcare services to its citizens [38]. The European Commission and the member states agree that it is now essential to provide a legal framework for digital health initiatives, so as to give them a real European dimension and establish a secure, stable, and interoperable framework for them, as a necessary condition for building a robust Europe of Health through digitalisation [27]. 

The EHDS has multiple ambitions—for individual citizens, for clinicians and physicians, and for research [39]. It is expected to help patients who currently cannot access a real continuum of care when they move within the European Union, owing to the differences among health systems in terms of funding and organisation of care, and in the face of the lack of information sharing between member states. They will be able to access and share their health data—what is known as primary use of data—and health professionals will be able to access care pathway documents in their original format, and translated into their language [1,19,20,21,27].

The regulation also aims to allow researchers, innovators, and policymakers to access health data in a uniform manner at the European level (the secondary use of health data), within a secure framework and in compliance with all EU legislation covering health, digital, or protection of citizens. Access to this data is currently subject to national divergences and delays, governed by the regulations of the member states, and conditional on a request to each national authority in accordance with its own procedures. European ambition should make it possible to remove these obstacles too, allowing their reuse under secure and specific conditions [28]. As health commissioner Kyriakides said in June 2022, “health data has vast potential to improve the health of our citizens if we manage to use it in an effective and safe way”, and “sharing data will save lives. EHDS will help innovation in healthcare, which is all the more crucial as new health threats arise”, she added. “A new vaccine or critical therapeutic may well arrive more quickly, because it will prove far easier to conduct research on a European scale with higher quality interoperable data” [29].

The EU Presidency has underlined the need to ensure citizens’ trust in digital health, which should therefore be based on appropriate security, interoperability, and ethics. So too has Kyriakides: “From the very beginning, the key word when working on this proposal was trust. We need to make sure that there is a climate of trust, where every citizen can be confident that their personal health data is handled with the greatest care, underpinned by very strong data protection rules and data security” [29]. European ethical principles for digital health have already been adopted unanimously by the member states within the framework of the eHealth Network [27]. 

## 3. Where Is the Opportunity?

There is much to be welcomed in this concept. Developing a governance framework specifically for a sector is something of a departure in EU policy terms, but is very much the right approach. It differs fundamentally from earlier approaches that have led to confusion—most notably the choice of separating healthcare from the EU services directive, but subsequently too in what has been in effect a retroactive approach, as with In Vitro Diagnostic Regulation (IVDR), the clinical trials regulation, or the medical device regulation, all of which have encountered difficulties in implementation.

The hope is that this proposal will mark a shift in developing EU legislation in the health field. It signals a move away from a pattern of impetuous legislation that later proves problematic at the implementation stage, requiring complex remedial measures and extended transition periods to accommodate challenges insufficiently considered at the outset—as exemplified by the repeated attempts to legislate on clinical trials, or the overhasty adoption of the far-reaching GDPR, and as will now be replicated in the Health Technology Regulation. It also offers the opportunity to the healthcare sector itself to demonstrate that it too has evolved to the stage where it can engage in mature dialogue with legislators to constructively shape the proposal [23,25].

Ideally, what will emerge from the discussions around this proposal over the next two years will establish norms for a more effective use of data, benefiting fully from the potential offered by technology and new methods. The alternative scenario of rigid and arbitrary limits on data storage or access, with so many safeguards that the object of the exercise is defeated, is, of course, less attractive. If legislators get it wrong, Europe could be condemned by the final form of the legislation to the/a perpetuation of traditional approaches, consolidating resistance to change even among clinicians wedded to handwritten notes, or among insurers tied rigidly to concepts of data they will accept, or among medical specialists resistant to new technology and regulators fixated on conservative judgements.

## 4. Where Are the Challenges?

So, what is at stake is whether Europe now is sufficiently ready and willing to construct and adopt an approach that respects the opportunities before it. The key lies in maximising a governance approach rather than a legislative approach, so as to ensure clarity and coherence, along with the flexibility to continue taking advantage of opportunities as they arise for integrating data into the broader picture of improved patient care and health provision for European citizens. The paper does not present a model, as the European Health Data Space is itself the model. It provides a policy discussion at the interface of science and policy with legal and healthcare systems.

The disappointing experience with GDPR, with its priority of protecting patients in an absolute and abstract way, is instructive [40,41]. This has displaced the possibility of understanding or coping with technological innovations offering solutions for practical or unmet healthcare needs that were unforeseen. Better results in translating innovation and empowering data to healthcare’s advantage will come from a governance framework that allows a rational approach and takes account of the characteristics and idiosyncrasies of different healthcare systems as well as of progress in technology and innovation [42].

The enthusiasm widely expressed at the idea of EHDS is also conditioned by a wide range of specific concerns, reflecting the distinct perspectives of the many stakeholders [25]. The welcome from the European Confederation of Pharmaceutical Entrepreneurs is subject to the reserve that the outcome should be “a fit-for-purpose regulatory framework.” Its top concerns are overcoming “disparate quality and complexity” in a “fragmented” European data landscape and tackling “member states’ diverse interpretations” of EU data protection rules. Medtech Europe shares this concern: EU legislators need to ensure that the EHDS regulation is in clear alignment with existing legislation, such as the Medical Devices Regulation, the In Vitro Diagnostic Medical Devices Regulation, the General Data Protection Regulation, and other recently proposed legislation (Data Act, AI Act, etc.). It says a consistent legal framework paired with clear and transparent rules on citizens’ access to their data will provide the right context for a trustworthy ecosystem that protects individuals’ rights and unlocks the potential of health data. EPF emphasises that the framework must be “shaped and implemented properly with patients at the centre,” since its success will depend on its ability to reach out to patients and citizens, and to be accepted by them. “Meaningful involvement” of patients in governance “should not be conditional on the topics discussed and their degree of sensitivity” [30]. EURORDIS adds to its welcome the insistence that EHDS must be accompanied by a European Commission commitment to improving digital health literacy: “Patients need to know their rights, especially those with rare diseases who often manage their data in a cross-border environment.” Digital Europe’s support is also conditioned by concerns that aspects of budget, governance, and interplay with other legislation will need to be clarified, and quickly too, if the necessary infrastructure is to be up and running in all member states by the envisaged date of 2030. It also wants greater clarity over funding and coordination as the system is constructed [32]. With so many differing views, the upcoming discussions are going to have to not only successfully navigate the evident technical and political challenges, but also to reconcile competing and occasionally conflicting positions of the parties to the discussions.

Of particular concern in the current proposal is the desire for creation of new constructs such as the right to be forgotten, strongly welcomed by patient associations [43]. While well-intended, this initiative could prove unhelpful to public trust, and even undermine it with the implication that public institutions or private healthcare actors such as insurers are not to be trusted. The creation of new legal regimes risks adding complications when existing antidiscrimination frameworks are already in existence and have a body of case law that supports patient protection. Perhaps more importantly, since it is widely accepted and reiterated that a successful EHDS depends on shared trust, there are balances to be achieved between overburdening the system with complications and responding properly to any lack—justified or not—of public trust.

But the key issue is ensuring adequate scope for re-use of data for research. The implementation of GDPR has shown that even when the spirit of law has the right trajectory—to ensure that data is protected—there can be devils in the detail, and GDPR, which suffers from the critical omission of an interpretation layer prior to implementation—has hampered innovation in healthcare as different member states, regions, and healthcare institutions have implemented the regulation in different ways [40,44,45].

## 5. The Detail

In the EHDS proposal, specific provisions govern both primary and secondary use of electronic health data [23]. New patient rights were created over the electronic health data alongside obligations on health professionals, with member states required to set up a digital health authority and designate a national point of contact. Other obligations in relation to primary use cover interoperability of health-related data sets, and the establishment of a common infrastructure to facilitate cross-border exchange of electronic health data, known as MyHealth@EU [46].

Specifically for secondary use, the legislation aims to facilitate this for research, innovation, policymaking, patient safety, or regulatory activities. It defines data types that can be used for identified purposes and sets out prohibited purposes. Additionally, it requires member states to set up a health data access body for secondary use to issue permits to data users. The purposes for which electronic health data can be processed for secondary use are spelled out in the regulation and include public interest in health protection against threats, surveillance, and ensuring high levels of quality and safety of healthcare and products [28,47].

There are also measures to promote capacity building by member states in relation, for instance, to the exchange of information on digital public services or funding, national access, and rules on international access to nonpersonal data. However, a key issue is the sheer heterogeneity of digitalisation maturity of member states, and indeed of healthcare provision per se. The EHDS legislation is not being proposed across a level playing field in the EU. The regulation will have a one-year transition period for most provisions and three years for provisions on electronic health record systems [28]. 

## 6. Discussion

The reality that confronts policymakers is the classic problem of knowledge limitation. On many of the issues raised by the proposal, the answer is known to be known. On many others, the problem is known, but the answer is still unknown. Legislators and decision makers feel they are on firm ground in both these scenarios: either a solution exists, or a solution can be sought. The real challenge is recognising that there are also unknown unknowns—and here the law of unintended consequences hovers menacingly. There is the risk of acting prematurely or deciding impetuously, without taking into account the possibility that legislation today could prove, tomorrow or the day after, to be more harmful than beneficial. The more that expert input from the scientific and research community feeds into the upcoming discussions, the better the chances of policy decisions that genuinely enhance the opportunities for the use of health data.

It is not an unprecedented challenge. The impact of multistakeholder views—articulated by EAPM in 2015 [48]—resulted in valuable and substantial policy changes endorsed in Council conclusions on personalised medicine for patients [49] that reflected the ambition of the United States Precision Medicine Initiative launched by Obama. Similarly, EAPM contributions to the 1 Million Genome Declaration via its MEGA initiative [50] helped improve data interoperability in that field. EHDS has the potential to extend that process across all data sites.

This lends additional significance to the EU’s discussions of its EHDS, as the legislation—which relates strictly to health data—could turn out to be a harbinger of the ways the EU intends to legislate in all areas of data (since it is planning to develop legislation in agriculture, manufacturing, energy, and half a dozen other sectors.). If the EU manages to create an optimal framework, it would be an asset for the use of data in general and data in other sectors. By the same token, if the EU fails to do so, the prospects are accordingly dim both for the health sector and for other sectors—as well as for the general principles that will be established in EU rulemaking [28]. 

### 6.1. Understanding the Issues

The underlying theme of the proposal is the bringing of innovation into healthcare systems, and discussing its content is inevitably anchored around that theme. However, another of the principal areas for discussion is the scope for data-sharing in the context of the earlier GDPR [40,44,51]. In addition, it is also impossible to explore the implications of the proposed framework without taking full account of the issues it raises of interoperability, among distinct physical systems, and also in relation to operational aspects of healthcare such as products—and particularly medical devices and IVDR—and processes such as clinical trials and authorisation of medicines.

The potential conflicts can emerge across a range of novel provisions. For patients, EHDS gives them new rights over their electronic health data and imposes obligations for using that data in primary care. That will have to be satisfactorily reconciled with rights and obligations under GDPR [28]. Data holders will be subject to new obligations to provide electronic health data for secondary use. Technology providers—of electronic health record systems, some medical devices, and AI systems—will face new obligations, including meeting premarket conformity assessment requirements, albeit self-certification. Additionally, digital health services and wellness apps will also face new rules. The introduction of a new and harmonised scheme for giving access to electronic health data will add further complexity for providers and users. Outstanding questions therefore include issues of definitions, rights, obligations, and technology.

In the proposal, the definition of personal electronic health data is “data concerning health and genetic data as defined in [the GDPR], as well as data referring to determinants of health, or data processed in relation to the provision of healthcare services, processed in an electronic form”. A list of the categories of personal electronic health data covered by the new rights is already lengthy and likely to be extended (currently, it covers patient summaries, electronic prescriptions, electronic dispensations, medical images and image reports, laboratory results, and discharge reports) [28]. 

The scope of potential enforcement and redress is also subject to some uncertainty: individuals’ requests for exercising their rights may be directed principally at healthcare hospitals and HCPs, but could be wider, even as far as clinical trial data, and all data users will have to make sure their medical devices and software allow for compliance. That, in turn, has implications for manufacturers and service providers. GDPR’s provisions on enforcement and penalties will apply, and EHDS itself makes provision for heavy administrative fines for noncompliance with individuals’ rights. Mandatory self-certification schemes will be set up for the (very broadly defined) EHR systems, along with postmarketing requirements and reporting obligations for serious incidents. Uncertainties also remain over the significance of related references in EHDS to “in the health system”, but the result could be legislation of very wide application, including to wellness apps. The interplay with MDR and IVDR also remains opaque at present, with much to be decided at a later date through implementing acts [27,28]. The definition of the term electronic health record (EHR) system is also very broad. This leaves unclear when an EHR system would qualify as a medical device, and what the implications would be for a device employing AI on the data. The legislative proposal appears to assume a possible demarcation between EHR systems, medical devices, and high-risk AI systems. If products or services fall under all categories, it could result that manufacturers could be mandated to conduct conformity assessments under different regulations. The interplay of legislation—including with the EU Data Act—leaves open the risk of a cumulative over-regulation of products and data.

For secondary use, data users must make applications to access bodies (that will, in most cases, be national gatekeepers), for a permit to access identified data sets. MS have to establish one or more of these Health Data Access Bodies—and there is a risk of fragmentation as this could also lead to deviating implementation and enforcement of the provisions. The permit will require compliance by the data holders (for which the definition is very extensive)—but the list of categories of data that must be supplied is extensive but still incomplete, and the limitation of how broad a data access request can be, or how far back in time it can go, is still unknown. It seems likely that electronic health data from clinical trials may fall into the category of data which must be available for access—but there is still considerable lack of clarity around these requirements.

There is also uncertainty over what measures access bodies will be required to take to preserve confidentiality when data requests cover electronic health data protected by intellectual property and trade secrets. There are also limits foreseen on transfers beyond the EEA of data deemed highly sensitive. The freedom of data users to process electronic health data they access is tightly limited by EHDS.

In addition, the degree of freedom given to member states in setting up their own access bodies carries the risk that divergent approaches may emerge at the national level, in contradiction to the aim of reducing fragmentation and incoherence in data legislation.

On systems and interoperability, compliance with and use of the dedicated MyHealth@EU infrastructure will be necessary for data transfer, with implications for systems supply. (At the time of writing, use of MyHealth@EU, or summary records, or other recommended mechanisms, is very low). The relationship between existing international data interoperability standards and possible new specifications dictated by the Commission creates another uncertainty [28].

The relationship between EHDR and GDPR will absorb much attention. The new rights conferred by EHDS do not prevent individuals from exercising their GDPR rights. Data users and the access body issuing a permit will be joint controllers for data processing in the meaning of the GDPR—and consequently liable for damages in the event of a breach. The transparency requirements under GDPR and EHDR overlap in some areas, and appear to diverge in others [28,44]. 

Similarly, it is yet to be established what significance is to be read into the EHDS provision that “Synergies between the EHDS, the European Open Science Cloud and the European Research Infrastructures should be ensured, as well as lessons learned from data sharing solutions developed under the European COVID-19 Data Platform” [28,52].

Stakeholder involvement is still something of an open question, with concerns that existing possibilities may not be sufficient or adequate for effective access to the European Health Data Space Board that will be set up as a governing body. Requirements for publishing the results of studies using secondary data contain some imprecision as to timetabling and scope. There is also still uncertainty over when EHDS will be adopted, and consequently for the timetable for subsequent implementation.

The interplay between EHDS and the draft Data Act will also require elucidation in relation to definitions of “products”, “related services”, EHR Systems, and “appliances”, as well as to resolve potential conflicts between their divergent definitions of “data holder” [19,28]. There is also a lack of clarity on the interplay between the two proposed Acts in relation to the protection of IP and trade secrets.

There are also uncertainties over the territorial scope of the rights conferred, and how far these are limited to the EU or EU citizens, the definition of “dataset catalogue”, and the anonymisation of datasets [28]. 

### 6.2. Clear Potential Benefits

The challenges should not lead to losing sight of the potential benefits. The EHDS framework for sharing electronic health data will make it easier for healthcare providers to have access to electronic health data from other healthcare providers about the patients they treat, and easier for universities to access quality data to carry out research. It explicitly provides that it will “support health research, innovation, policy-making, regulatory purposes and personalised medicine purposes, and also aims to make it easier to provide cross-border healthcare”. Indirectly, it could also result in more collaborations between industry and academic institutions and researchers on investigator-initiated studies. The draft proposal claims that EHDS “will create a legal and technical environment that will support the development of innovative medicinal products and vaccines, and of medical devices and in-vitro diagnostics” [28]. 

### 6.3. Views from the Ground

The reality of data sharing is not an academic or political exercise conducted in discussions of theory. The reality is the countless individual researchers and organisations that are generating, collecting, analysing, and making use of health data—or that know they could do more if they had better access. Sectors and organisations with a stake in the generation collection and use of health data have their own takes on the proposals, based on their own specific perspectives.

EATRIS, which specialises in translational research [53], notes that the proposal rightly tries to address the challenge that GDPR has created for secondary use of data across the EU, but is concerned that the permit-based system, which would seem to support the Federated model of data access, may be unduly bureaucratic. It also perceives a potential conflict that the proposal seems to set EHDS as a legal basis to access health data in compliance with GDPR. On consent, EATRIS favours opt out of consent in processing of data for secondary use and establishment of competence centres for secondary data management for research, to ensure safety certification, a digital research environment, responsibility, data analysis design, and interpretation of skills.

For the European Heart Network, concerns focus on privacy and data security, lack of accessibility, lack of personal motivation, and low digital health literacy. What it wants to see is greater transparency, equal access, digital health literacy, meaningful patient and public involvement in data intensive research and innovation, interoperability with data standards, and quality controls.

For Juvenile Diabetes Research Foundation (JDRF), a private diabetes research foundation [54], there is a wealth of data available in population-based registries, national audits and surveillance systems, national databases for quality indicators, and different types and levels of data sources including academic and regional. However, much of this information, even where it is of high quality, is heterogeneous, fragmented, not based on standardised measures, lacking solid international comparisons, difficult to connect, covered by different principles for data sharing, and available normally only in national languages. The right sort of legislation could improve health provider communication, help physicians and patients make shared decisions, and allow patients to travel with the data. In hospitals, this could allow providers to measure what they achieve and compare, and it could facilitate benchmarking on data and outcomes at the broadest level of health and social care. It could help the design of better payment models and redefine what is an ideal quality of care. However, for the research community, its principal added value would be to improve the quantity and speed of research studies, accelerate research towards finding a cure, and accelerate research towards effective prevention. It could give new significance to real-world evidence and patient-related data and could be mobilised in risk stratification and personalised care models. It is, in short, a great opportunity, but the central need is to integrate Europe’s existing data sources as well as new sources of data. Implementation strategies for using data and results in clinical practice should take account of international examples.

In prostate cancer (PCa), where the need is evident for integration of real-world clinical data into disease classification and care pathways, the lack of standardisation of PCa-related outcomes leads to the lack of appropriate patient stratification, insufficient engagement of stakeholders including patients, and suboptimal care [55]. The PIONEER network of 34 public and private stakeholders who collaborate closely with internal and external data providers to build a prostate cancer big data platform faces major knowledge gaps [56]. 

The increasing abundance of real-world evidence is another opportunity that is not being realised because it is still not widely accepted by regulators [42,57]. Most of the focus is still on the methodological and quality aspects of the data, but accessibility is in itself a major barrier for all stakeholders. A solution requires an exploration of who stores the data and with what safety levels, whether the data can be shared and in which format, where the are data used, for what purpose, and with what level of active patient consent, and who owns the data [58,59].

MedTech Europe highlighted the need for a health data ecosystem that fosters trust and protects individuals’ rights while unlocking the great potential of health data. The new legislation should address existing legal and technical barriers to data sharing and advance investments in technical infrastructure and the upskilling of the digital health workforce. Fostering ground for the adoption of internationally recognised interoperability standards will also be crucial. The medical technology industry underlined that a successful EHDS will depend on clear rules that are aligned with existing legislation, and should support effective access to health data, to allow for better research and technology innovation.

From a public health perspective, the proposal has implications for precision medicine, in terms of family studies, studies estimating the contribution of genetic in the environment to healthcare cost and comorbidity, genetic studies using biobank data sets to explore associations between new genetic variants and novel risk factors of disease, public health predictive studies such as real-time surveillance systems or detection of disease outbreaks, population health studies to identify high-risk behaviours, and learning healthcare systems [28].

A project aimed at early diagnosis of rare disease in newborns through genetic screening and artificial intelligence using machine learning for health data, Screen4Care, aims to screen asymptomatic newborns, explore next-generation sequencing, expand the spectrum of rare diseases included in screening, and use whole genome sequencing in early symptomatic infants. However, there are immense ethical complexities in defining actionable diseases, and because science and technology are continually changing, any list would have to be dynamic [60]. 

## 7. Conclusions

Europe has an opportunity here to bring its health policy closer to current—and crucially to future—needs and possibilities. An effective framework can be built within the framework proposed by EHDS. Europe also has the chance here to establish international leadership in the field, as interest in digital health is currently growing in G7 and G20 fora, and will lead, in time, to action globally [27]. 

In the real world, there is no possibility of 20/20 foresight, and no prospect of omniscience—so decisions have to be taken, even based on imperfect knowledge. However, what can mitigate the risks of trouble further down the line is to ensure reflecting with due caution and consideration on possible implications and weighing in carefully the competing and possibly conflicting inputs to a debate.

The GDPR has been a good lesson of what works and does not work in terms of thinking ahead on decision-making [41,45,51]. The European Parliament discussions reflected democracy in action in that it led to over 4000 amendments to the proposal—but the outcome has left considerable weaknesses in the EU, particularly as so many national divergences in its application still exist—with negative consequences for many activities, including research [61]. High-quality input to the debate at its formative stage can help to reduce the risks of such a fragmented and confused decision-making process recurring with EHDS.

The EAPM expert panel concluded that deriving the full benefits of the European Health Data Space will depend as never before on Europe making a conscious decision to act in concert, with the right legislation and the right policy decisions, so as to take the chance that is on offer. Success requires that data can flow more freely, rather than being trapped in traditional silos or constrained by artificial borders. Success also depends on something less tangible: readiness by everyone involved to make use of the data that will become available. This will require buy-in by EU and national policymakers, by health authorities at all levels, and by stakeholders—patients and citizens—who stand to gain most, and who are also determinant in how far further benefits will be conferred on future generations of Europeans. Individuals are both the sources for and the beneficiaries of this wealth of data. The appropriate use of new technologies and techniques—notably genomics and genomic data—will maximise the benefit to health systems and patient care. Additionally, for once, it is individuals who are in a position to influence the outcome. In very real terms, this time the future is in their hands.

The short answer therefore is that Europe is able—in principle—to seize the chance for wide healthcare progress. However, its willingness needs the input of all stakeholders. Taking the opportunity will require a readiness on all sides of the European debate to perceive the true potential of health data as an asset with investment (i.e., not just a cost of operations), and to honestly and pragmatically work towards creating an effective framework for capturing and deploying it to the advantage of patients, public health, and European quality of life. The familiar concept of ‘from bench to bedside’ in translational innovation remains as valid as ever, but the key issue here is more a question of data “from bedside to bench and back to bedside”. Without providing conditions now for making better use of the vast sums of health data already in existence, and the even greater quantities that emerge as science and technology advance, the potential benefits will not be realised. It is a loss that Europe and its citizens cannot afford, and failure now will be seen in the not-too-distant future as an unforgivable breach of duty among policymakers and the stakeholders involved, as well as weakening Europe’s international competitiveness. As Kyriakides has said, “We have already laid the groundwork for the European Health Data Space. If we focus our efforts together, we can start to truly unlock the benefits of the digital transformation for our societies” [29]. 

## Data Availability

Not applicable.
